# Women’s Perception of Spousal Psychotic Disorders: A Qualitative Study

**Published:** 2019-10

**Authors:** Mohsen Koushan, Nematullah Shomoossi, Zahra Parsaei Mehr, Mostafa Rad

**Affiliations:** 1 School of Nursing, Sabzevar University of Medical Sciences, Sabzevar, Iran.; 2 School of Medicine, Sabzevar University of Medical Sciences, Sabzevar, Iran.; 3 School of Nursing, Iranian Research Center on Healthy Aging, Sabzevar University of Medical Sciences, Sabzevar, Iran.

**Keywords:** *Psychotic Disorders*, *Qualitative Research*, *Spouse*, *Women*

## Abstract

**Objective** **:** Mental disorders are often accompanied by serious problems in personal and social communication, including marital problems. In this regard, women whose spouses have mental disorders are less likely to express their mental experiences and emotions due to the cultural barriers of the community. This study aimed to identify and explain the shared mental experiences of women whose spouses suffered from psychotic disorders.

**Method** **:** This was a qualitative study with a content analysis approach. In total, 15 women whose spouses were admitted to the psychiatric ward of a hospital due to psychotic disorders were selected through purposive sampling. Data were collected by conducting in-depth semi-structured interviews on experiences of women regarding their spouses’ psychotic disorder. Data were analyzed using conventional content analysis techniques.

**Results: **In this study, 28 subcategories and 7 main categories (lack of intimacy, social constraints, dual emotions, confusion and mental exhaustion, fear and concern, coping strategies, and life problems) were developed.

**Conclusion: **Based on the results of the study, women whose spouses suffered from psychotic disorders experienced various psychological challenges. Therefore, it is recommended that the health care staff take such psychological challenges into consideration to design and implement effective strategies to solve the related problems.

The spouses of the psychotic patients appear to be suffering from considerable levels of stress for various reasons (1). Because of the lower satisfaction levels in marital needs, on the one hand, and witnessing the moment-by-moment suffering of their loved ones (diagnosed with mental illnesses) and not being able to provide for the family and treatment of their husbands, on the other, these women are doomed to endure severe and considerable stress (2). In addition to causing severe disability for individuals and disrupting their social and individual functions, mental disorders, particularly psychotic disorders, cause difficulties in fulfilling marital and family duties (3). Studies have shown that the development of mental illness in a family member imposes considerable stress, concern, and negative consequences on the family (4). In addition, evidence suggests that the spouse of a person with mental disorder experiences moderate stress and that caring for the-patient brings about more negative impacts on their mental health (5).

The results of a study by Von Kardorff et al revealed 11 upsetting themes in the caregivers of patients with psychotic disorders: 1- instability of life, 2- lack of awareness, 3- emotional load, shame and stigma, 4- financial burden, 5- physical load, 6- limitation in life routine , 7- disruption in life routine, 8- family dissatisfaction, 9- relatives of acquaintances, 10- problems related to drug treatment of the patient, and 11- problems related to health services and government support (6). Therefore, in addition to marital problems due to the illness of the spouse, women must care for their spouse who suffers from mental problems. Moreover, frequent admissions during psychological attacks and financial problems imposed on the family due to the job loss of the spouse can cause physical, psychological, financial, and social distress for the family (7, 8). 

The care of a person with mental illness affects the caregiver both emotionally and physically, leading to family conflicts (9).

Because of the high level of stress and intolerance of many life problems, these women may also be less able to maintain previous friendships or create new friendships (10). They are involved in such stressful events that they should also be considered as patients to prevent additional conflicts (11). In cultural context of Iran, women with sick spouses often remain married and carry the burden of caring for the patient and providing family expenses along with the experience of a failed marital relationship. Therefore, great attention should be paid to these individuals, and information must be obtained about their mental preoccupations, daily problems, and their sorrow and sadness. Furthermore, a review of the literature has revealed that little research has been conducted on women's experiences of living with spouses diagnosed with and suffering from mental illnesses, especially in Iran. In addition, such problems and complications bear unique essence across different societies and cultures, where its evaluation becomes essential. Therefore, conducting a qualitative study seems to help explore unique insights into women's perceptions of living with a lifelong partner suffering from mental illnesses. Therefore, the present study aimed to explore and describe the experiences of women about living with spouses suffering from psychotic disorders. In other words, the present study aimed to evaluate the lived experiences of women about the diagnosis of psychotic disorders of their spouses, including their emotions and behavioral reactions to the situation.

## Materials and Methods

***Design***

Given the objective of this study, which was discovering the mental experiences of wives of men with psychotic disorders, a qualitative approach was used to comprehend and describe human experiences and ascertain hidden meanings in their daily experiences. This research method is mostly applied to extract the main themes and to discover patterns in the data. In addition, it is a proper technique for achieving valid and dynamic results affected by a deep understanding of the data field to shape the new knowledge and provide reality and clinical guidance in practice. In fact, this method attempts to understand the meanings in the data by qualitative reduction and data classification (12).

***Participants***

In this study, the participants included 15 women whose spouses were diagnosed with severe psychotic disorders and were admitted to the psychiatric ward of Vasei hospital in Sabzevar, Iran. A total of 15 women were selected by targeted sampling. Maximum variation was used to select the participants, and women with varying duration of marriage (2-30 years) were enrolled in the research. Moreover, the husbands suffered from different types of mental illnesses, ranging from severe depression to schizophrenia. 

In addition, the participants had different levels of education and occupations. Sampling continued to data saturation with the maximum variety, meaning that more interviews did not lead to obtaining new information for the development of new categories of women’s experiences. In other words, no new data were obtained in the last three interviews. This study was conducted during March and September 2018. Inclusion criteria were at least 2 years of marriage, willingness to participate in the study, being able to express the experiences, and age range of 23-57 years. In qualitative research, the number of samples is not constant and sampling continues until no new idea is obtained (13).

At first, the second author explained the objectives of the study to the participants and ensured them of the confidentiality of their personal information. Afterwards, the interviews started with warm-up questions about the demographic characteristics of the participants, including age, history of marriage, occupational status, level of education, illness of the spouse, occupational status of the spouse, and number of children. Then, the interviews continued with a general question about their understanding of living with a mental patient. More detailed questions were gradually asked, such as one’s feelings in high-pressure situations, reactions to life tensions, and how and why the person still continues to live in this situation. If necessary, more exploratory questions were asked such as “Please elaborate on that”, “What happened next?”, “What preoccupied your mind?”, and “How did you feel then?” In each session, the interview ended with two questions: “Was there another question to be asked? or “Do you have a question from me?” Data were collected through in-depth and semi-structured interviews regarding the experiences of women about living with a mental patient. Each interview session lasted for about 60-90 minutes. Also, to obtain further information, 3 of the participants were invited to the second round of the interviews. 

***Data Analysis***

After repeatedly listening, transcribing and analyzing the data, another time was arranged for an interview in case further information was needed. The time and place of interviews were determined based on agreements between the participants and the interviewer. The interviews were held in the interview room at Vasei hospital in city of Sabzevar, where the spouses of patients had come for a visit. The interviews were recorded after obtaining consent from the participants. Next, the researcher listened to the interviews several times and transcribed all of them on paper. In the next stage, each interview was analyzed, and the next interviews were performed after precise evaluation. Data analysis was used to analyze the qualitative content applied for subjective interpretation of textual data. 

After listening, data mining, and reading the data, a general image of the data was obtained, and the meanings were extracted. Moreover, the key ideas were highlighted, and the codes were classified based on their relationship with each other. The validity of the data was confirmed using various strategies, including peer-check techniques and member check. In the evaluation of participants, the interview codes were returned to the participants who confirmed the codes extracted by the researcher. In the peer-evaluation technique, two researchers performed coding and classifying stages separately. Then, a discussion was held to reach consensus in case of lack of agreement on coding and categorizing. In addition, the MAXQDA10 software was exploited for more efficient analysis, maintaining, and organizing the data.

## Results

A total of 15 women aged 18–55 years participated in this study. Their marriage duration ranged from 2 to 30 years. Major types of spousal disease were schizophrenia, manic depression, and severe depression. On average, the time of disease diagnosis dated back to the past 8 years. 

After data analysis, 800 primary codes, 28 subcategories, and 7 main categories were developed. In this study, the 7 main categories included lack of intimacy, social constraints, dual emotions, confusion and mental exhaustion, fear and concern, coping strategies, and life problems. Each category included some subcategories, which are presented in Box 1.

The experiences of women participating in this study were indicative of modes of emotional deficiency, inability to freely express feelings, feeling of loneliness at home, and lack of physical intimacy. 

***Lack of Intimacy***

***Emotional deficiencies***

Experiences of women showed their extreme emotional deficiency. Lack of physical intimacy such as sleeping in separate beds and decreased sexual relations or discontinuation of sexual intercourse stated by participants.

“We have a sexual relationship, but it is much less frequent than before. In the past, we were very intimate but none of that exists today.” (12th participant)

***Feeling of Loneliness at Home***

The feeling of loneliness with signs of inadequate spousal support, feeling of emotional failure, and inability to accept affection from one’s spouse due to hatred, origination from his earlier words and behavior over time were other experiences expressed by participants. 

“….He showed no emotion and had no feelings towards me. He never said, “I love you”. I feel so desperate and alone.” (First participant)

***Unable to Freely Express Feelings***

Women do not experience a sense of calmness when they are with their spouse but cannot make verbal and nonverbal communication. This has led to a sense of inability to freely express feelings.

 “….But he would say, leave me alone, you are so clingy. I was so disappointed and heartbroken.” (Sixth participant)

***Social Constraints***

***Feeling of Others Distancing Themselves***

The negative experiences of women about the mental illness of their spouses included a feeling of others distancing themselves from them; and this negative attitude led to their isolation. For instance, the mental status of the spouse and concerns about others’ behaviors and their possible negative attitudes, reduced the desire of the participants to go to parties. 

 “I cannot go anywhere with him, because I am humiliated by my family and relatives.” (Fifth participant)

***Feeling Ashamed of the Behavior of the Spouse***

Most participants expressed that they were ashamed and afraid of the illness of their spouse and felt abandoned, which led to fewer social interactions.

“My husband shows bizarre behaviors or uses vulgar language, so I am embarrassed to participate in parties and gatherings.” (11th participant)

***Dual Emotions***

Women participating in this study described dual emotions, such as a feeling of suffering vs tolerating the difficulties, feeling of sympathy vs feeling of hatred, and feeling of responsibility vs feeling of regret. 

***Suffering/ Tolerating the Difficulties***

 The experiences of the women were indicative of their suffering in living with a mental patient. Most men with mental illnesses tended to disrespect their wives and families. Some of the issues that caused the suffering of women in living with a mental patient included beating, ill-temper, beating of children, sexual harassment and forced sexual relations, unemployment leading their wives to seek jobs out of home, insulting, pessimism, accusing their wives of disloyalty, and creating discomfort and crying. From another perspective, the experiences of women showed their tolerance of many difficulties in living with a psychotic patient, in a way that women were led to take the responsibility for their family and to suffer a lot of hardship, such as unemployment of their spouse, difficulty in finding a job; they also suffered the stigmatized difficulty of a female working in a traditional society, and tolerating unpleasant behaviors of the spouse and the situation hoping that the condition of the spouse could be improved.

“He refused to take care of our children. I always took care of them myself; at the same time, I worked in the winter and summer. It was really hard for me to see him stay at home while I go to work.” (10th participant) 

***Hatred/ Feeling of Sympathy***

 One of the feelings expressed by women as their experience of living with this type of men was hatred. Hard situations such as neglecting and beating one’s wife and accusing them of disloyalty increased nothing but hatred towards one’s spouse, to the extent that they would develop a tendency to commit suicide or wish the death of their spouse. Instead, these women developed a sense of sympathy toward their spouses: “I really felt sorry for him.” (Eighth participant)

“….I hated him since my daughter was born. I do not want to be with him or even walk with him in public.” (Ninth participant)

***Regret/Acceptance of Responsibility***

The experiences of women were also indicative of their regret for marrying their spouse; however, they expressed that regardless of their unhappiness, they would do their best to improve the disease of their spouse and decrease his mental problems. “I regret being his wife. However, I will properly raise my children and try to make him better.” (13th participant)

***Confusion and Mental Exhaustion***

The experiences of the women showed their feelings of depression, guilt, and helplessness. 

***Crying and Feeling of Depression***

The participants also expressed that they were not happy and they would cry at the slightest things. They felt depressed and often tended to commit suicide. In addition, they wished death for themselves and their spouses. Due to several conflicts at home, children also showed aggressive behaviors, and the mothers did not tolerate conflicts between the children and treated their children harshly in such situations. 

 “I am always frustrated. I do not feel good. I have become depressed … and cry for a long time.” (Third participant)

***Feeling of Guilt***

The women expressed their feeling of guilt, in a way that they thought if they had paid more attention to selecting the right spouse or managed to establish a more reasonable life, this would not have happened to them. In addition, they felt guilty about the problems they had with their spouse, and they thought that they had sinned and their psychotic husband was the result of their sin. 

“… I must have taken him to the doctor sooner, but I did not. I could have borrowed money from others to do it.” (Sixth participant)

***Feeling of Helplessness***

Feelings of frustration and distress due to the current situation included consecutive hospitalizations and complications at home. The participants felt weakened and thought that their strength and tolerance were deteriorated due to the problems caused by their spouse. They lost hope for a good life and were willing to get a divorce. In addition, their spouses’ periodic accusations and misbehaviors reduced their capacity to continue their life and they felt a sense of failure. 

“I feel tired and at the end of my rope because of having an ill husband, visiting the doctors in hospitals all the time, feeling that my husband will never get better ….” (10th participant)

***Fear and Concern***

Fear and concern were also expressed by women, such as the fear of worsening of their spouse’s illness, of being harmed by the spouse, of future of children, and of divorce. 

***Fear of Hazardous Behavior of the Spouse toward Himself and the Wife***

Fear was another experience expressed by the participants, which were as follow: threats to burn the house or the wife and home furniture; murder in case of getting a divorce; suicide; and fear of being strangled by the spouse during sleep. 

“…He threatened me to kill my parents and then myself if we got separated. I was really afraid of him when he talked like that.” (11th participant)

***Fear of Divorce***

The participants also expressed that despite many problems with their spouse, they were afraid of divorce. They feared of being left alone and not supported by parents or brothers. Another issue was the problems due to divorce in Iran. Moreover, these women feared divorce because of their love for their children and concern about their unpredictable future. 

“I have no supporter. I have no one. I have a brother who is never there for me and I have a mother who can only feel sorry for me. … How can I get a divorce?” (14th participant)

***Fear of Worsening of the Mental Illness of the Spouse***

The fear of worsening of the illness was another concern. The participants realized that worsening of the menral illness of the spouse would be associated with a higher possibility of beating, insults, disgrace; and the fear never alleviates.

“Whenever his condition gets worse, I would think to myself that what would happen if we went to the park. For example, he would point to other women and ask me to check them out, or we would walk and he would say look at my wife, give her advice…. And at that point, I would die of embarrassment.” (Forth participant)

***Coping Strategies***

According to the participants, women living with psychotic patients used coping strategies to deal with the situation. In this regard, some of the strategies are emotion-focused and some other are problem-focused.

***Emotion-focused Coping***

The participants used emotion-focused coping strategies, such as ‘praying to God’, to cope with the situation. They also attended Quran-citing sessions to remain calm. Some of them believed that the hardship and torment in the world could diminish their eternal torment and hoped for a divine reward in the hereafter.

“… What could I do? I prayed to God day and night for his wellness.” (Ninth participant)

***Problem-focused Coping***

The experiences of the participants demonstrated that they did not deny any efforts to solve the psychotic problem of their spouse. In fact, they dedicated extensive efforts to the improvement of their spouse. In addition, emotional care for the spouse increased in some of the participants. One of the problems of these women was their husbands’ refusal to take the medications, for which various methods were applied to help solve this problem. Moreover, they tried to calm their spouses down whenever they became aggressive and angry. Consultation with a specialist and hospitalization are some of the problem-focused approaches in this respect. 

 “… whenever he started talking, I was able to realize that he had already stopped taking his medication. Sometimes, I would dissolve his medications in herbal teas, such as viper's bugloss and valerian.” (11th participant)

***Life Problems***

***Financial Problems***

A decline in the occupational performance of the spouse due to increased severity of the illness, lack of financial support by the family, especially the family of the spouse, women working outside home, and lack of a suitable job for earning a living were among the problems expressed by these women. 

“Another important issue is money. He is depressed, and I want to make him better. He must take his medications. I work and tolerate all problems.” (Eighth participant)

***Unemployment of the Spouses***

These women's experiences also indicated that their spouses were fired from their workplace after a period of conflict or depression due to their psychological problems. Therefore, their source of income was severely affected, and they had become poor.

“He does not work. He sits at home and does not work and his medical expenses are paid by Imam Khomeini Relief Foundation. It has been 6 years since he has stopped working.” (Ninth participant)

**Table 1:** Shared Mental Experiences of Women whose Spouses Suffered from Psychotic Disorders: Categories and Subcategories

1. Lack of intimacy
1.1. Emotional deficiencies 1.2. Feeling of loneliness at home 1.3. Inability to freely express feelingw
2. Social constraints
2.1. Feeling of others distancing themselves 2.2. Feeling ashamed of the behavior of the spouse
3. Dual emotions
3.1. Suffering/ Tolerating the difficulties 3.2. Hatred/ Feeling of sympathy 3.3. Feeling of regret/ Acceptance of responsibility
4.Confusion and mental exhaustion
4.1. Crying and depression 4.2. Feeling guilty 4.3. Feeling of helplessness
5. Fear and concern
5.1. Fear of hazardous behavior of the spouse toward himself and the wife 5.2. Fear of divorce 5.3. Fear of worsening of the illness of the spouse
6. Coping strategies
6.1. Emotion-focused coping 6.2. Problem-focused coping
7. Life problems
7.1. Financial problems 7.2. Unemployment of the spouse

## Discussion

Lack of sympathy, social constraints, dual emotions, depression and mental exhaustion, fear and concern, coping strategies, and life problems were categories derived from the experiences of the participants who lived with psychotic patients. In line with this result, Möller-Leimkühler et al showed that psychotic disorders led to disruption in the individual, social, family, and occupational performance. They also indicated that given the fact that these patients experience agitation and confusion of emotions and perceptions, their spouses need to deal with psychological problems in addition to tolerating financial problems and medical expenses (14). Also, studies have shown that the experiences of individuals diagnosed with schizophrenia, bipolar disorder, and depression affect the mental health of their spouses (15). According to the results of the present study, negative mental experiences of women living with a spouse who is diagnosed with mental disorders threaten their mental and even physical health. Therefore, diagnosis of mental problems in one of the spouses affects all family members, especially one’s spouse, laying the foundation for the possibility of depression in them. 

One of the important findings of this study was the lack of intimacy and emotional deficiency, which led to the feeling of loneliness. The participants suffered from emotional deficiency due to various reasons, including the addiction and illness of their spouse. Because of mental disorders and confusion of emotions, the men were unable to express their love for their wives. Social constraints were another set of experiences reported by the participants, which is incongruent with the results obtained by Karamlou et al. In fact, women living with mental patients experience issues in the society that limit their presence in the community. Shame and fear of judgment by others are among the major negative experiences in this regard. These experiences negatively affect one’s consulting a psychiatrist and following-up the illness, which ultimately results in the worsened prognosis and possibility of recurrence. Moreover, it affects the physical and mental health of women as a stressful factor (16).

In addition, the participants developed a feeling of hatred and regret of living a good life on the one hand, and felt sorry for their spouses and tried to help them recover on the other. Moreover, they complained about the behavior of their spouse but were not willing to get a divorce despite being constantly motivated to be separated from their spouse by their parents, family, and friends. Such reflections showed the development of dual emotions in the participants. In this regard, Scandberg et al described the everyday life experiences of relatives of individuals with depression, demonstrating that the main category of “living in conditions of change” was indicative of a significant challenge for the family. They reported that individuals with depression required balance in self-care; in their research, three descriptive categories were identified: ambivalent relation, regulation of daily life, and management of the situation (17).

Other aspects, including impatience, crying, feeling of depression, irritability, feeling of confusion, and helplessness, were among the experiences of participants derived from the interviews. These negative experiences were indicative of a type of confusion and mental exhaustion. In line with this result, Navidian et al found that most of care-givers of psychiatric patients, mostly women, suffered from mental exhaustion (18). Also, most of patients with mental illnesses are treated at home, and taking care of the spouse is often the responsibility of women. Providing long-term care to these patients can be harmful to women who are more vulnerable. One of the disorders diagnosed in these women is depression. In addition, feelings of guilt, incompetence, anxiety, anger, and aggression were reported by these individuals (17).

Furthermore, in a study by McAuliffe et al, evaluation of the experience of parents in caring for their children diagnosed with an illness showed their severe psychological distress when their children were diagnosed with schizophrenia. After this period, parents adopted a new caring role based on love and responsibility toward the treatment of their children (7). Four themes were extracted in their study, including psychological tsunami, care-providing activities, compatibility with permanent illness, and uncertain path. After a profound loss of experience, they moved on to accepting the problem (7).

Finally, fears and concerns were other notable concepts in the participants. In line with this finding, studies have shown that living with a person diagnosed with cognitive and behavioral disorders is associated with fears and concerns found in the statements of the participants such as the fear of being harmed and fear of the future (19, 20). The participants of this study used solutions to deal with stressful situations and ultimately maintain their own psychological balance. Along with attempting to treat the illness of the spouse, these women applied emotion-focused strategies, including praying or participating in religious ceremonies, which kept them calm.

## Limitation

The qualitative design of this study made it hard to generalize its results. Rather, it was attempted to investigate the data obtained by in-depth qualitative interviews, which reflected the participants’ perception of having a psychotic spouse. Another limitation of the present study was that the interviews were done only with women, while no interviews with the psychiatric patients were conducted. However, one of the strengths of the study was purposive sampling with maximum diversity that enabled the researchers to access a wider range of experiences as reported by the participants.

## Conclusion

According to the results of this study, women whose spouses are diagnosed with a psychotic disorder enter a challenging life. One of these challenges was the emergence of avoidance-avoidance conflict, meaning that on the one hand, getting a divorce would be associated with unpleasant consequences, and on the other, living and interacting with someone who is experiencing mental, emotional, and behavioral disorders is extremely difficult. Another challenge is the emergence of various psychological problems, such as irritability, depression, anxiety, feeling of confusion, and helplessness. In addition to affecting the quality of life of the person, these problems cause problems in performing maternal duties as well. Moreover, in the present study, it was determined that these women played the main role in providing care to their spouses without the help of others. Therefore, they are prone to poor mental health, which leads to their inability to solve family problems and caring for the spouse. Therefore, a defective cycle is created: the illness of the spouse leads to problems and challenges that affect the mental health of women; a weakened mental health leads to deteriorated performance of women in solving problems and caring for the spouse; the unwanted outcome of which is the increased burden of problems and aggravated condition of the patient; and a more severe illness in the spouse increases problems and challenges of the family. This cycle constantly continues. 

Therefore, each of concepts explored and emerged from the present data such as social constraints, dual emotions, fears and concerns, and copping strategies that included different dimensions of women’s psychological problems are suggested to be studied both qualitatively and quantitatively. In addition, researchers may also design other models to further explore these problems or to develop new strategies to help families, societies, and health care teams provide support to women whose spouses suffer from psychotic disorders.
